# The Regulatory Mechanisms and Therapeutic Potential of MicroRNAs: From Chronic Pain to Morphine Tolerance

**DOI:** 10.3389/fnmol.2018.00080

**Published:** 2018-03-16

**Authors:** Zhao Dai, Haichen Chu, Jiahai Ma, Ying Yan, Xueying Zhang, Yongxin Liang

**Affiliations:** ^1^Department of Anesthesiology, The Affiliated Hospital of Qingdao University, Qingdao, China; ^2^Department of Anesthesiology, The Affiliated Yantai Yuhuangding Hospital, Qingdao University, Qingdao, China

**Keywords:** microRNA, chronic pain, morphine tolerance, microglia, bone cancer pain

## Abstract

Chronic pain, including cancer-related pain, is a pain condition often caused by inflammation or dysfunctional nerves. Chronic pain treatment poses a significant health care challenge, where opioids especially morphine are widely used and patients often develop tolerance over time with aggravated pain. microRNA (miRNA) is known to play important roles in regulating gene expressions in the nervous system to affect neuronal network plasticity related to algogenesis and the developing of morphine tolerance. In this article, we reviewed studies conducted in rodent animal models investigating the mechanisms of miRNAs regulation in chronic pain with different phenotypes and morphine tolerance. In addition, the potential of targeting miRNAs for chronic pain and morphine tolerance treatment is also reviewed. Finally, we point out the directions of the future research in chronic pain and morphine tolerance.

## Introduction

Chronic pain is usually caused by inflammation or damage to the tissues or nerves in the nervous system. It becomes a major challenge in clinic with worldwide incidence of 20–25% (Breivik et al., 2006). Due to the limited understanding on the mechanisms of chronic pain origination, current available treatment options are often not very effective. Treatment with opioids for cancer-related pain and chronic nonmalignant pain (CNMP) (Rosenblum et al., 2008; Mercadante, 2010) often causes side effects such as morphine tolerance, which exacerbates chronic pain over time.

MicroRNA (miRNA) is small non-coding functional RNA that negatively regulates multiple gene expressions. The sequences of miRNAs and their target sites in mRNAs are extensively conserved across species (Friedman et al., 2009). Recent studies show that miRNAs play critical roles in the development and pathophysiology of the nervous system (Bhalala et al., 2013; Follert et al., 2014; Sun and Shi, 2015). Certain miRNAs are capable of regulating MOR expression (Wu et al., 2009, 2013). Conversely, MOR agonists like morphine and fentanyl also have been reported to modulate miRNA expression (Wang et al., 1993; Wu et al., 2008; Zheng et al., 2010).

The most widely used molecules to increase and decrease of specific miRNAs are miRNA mimics and miRNA inhibitors. miRNA mimics are double stranded RNA oligonucleotides that perfectly mimic the mature miRNA duplex. The guide strand targets mRNAs while the passenger strand is degraded. While miRNA inhibitors are single stranded oligonucleotides either of RNA or DNA chemistry, fully complementary to the miRNA to be silenced.

In this article, we summarize the findings from literatures investigating the mechanisms of miRNAs regulation in chronic pain and morphine tolerance. We also review the potential of using miRNAs as therapeutic targets and diagnostic tools from chronic pain management to morphine tolerance.

## MicroRNAs biogenesis and function

The biogenesis of miRNA can be summarized as follows: (a) RNA polymerase II transcripts miRNA genes to pri-miRNA (Yi et al., 2003). (b) pri-miRNA is cleaved by Drosha to become pre-miRNA and transported out of the nucleus by Exportin-5 (Yi et al., 2003). (c) Dicer and auxiliary proteins generates the mature double-stranded (guide strand and passenger strand) miRNA–miRNA^*^ duplex. (d) Ago2 and TRBP proteins incorporates the guide strand into the RISC complex, and the passenger strand is degraded (Khvorova et al., 2003).

The active miRNA–RISC complex then binds to one or more complementary sequences in the 3′ untranslated region (3′ UTR) of specific-target mRNAs to degrade or destabilize the target mRNA to inhibit protein translation. Each miRNA might target several mRNAs and a single mRNA can also be targeted by several miRNAs (Bentwich et al., 2005).

## The process from chronic pain to morphine tolerance

Chronic pain mostly arises from inflammation and tissue or nerve injury. Other incidents such as damage to the somatosensory system, cancer and genetic diseases can also cause chronic pain (Dworkin et al., 2013). Original stimulations are received by primary afferents and transmitted to the primary sensory neurons such as dorsal root ganglion (DRG) or trigeminal ganglion (TG) neurons. Then the primary sensory neurons transduce the stimuli into electrical signals which are transmitted to the spinal dorsal horn subsequently. In the spinal dorsal horn, the nociceptive and non-nociceptive signals are integrated and processed through complex circuits including excitatory and inhibitory interneurons, descending axons of brainstem neurons and glial cells (Kuner, 2010; Todd, 2010).

In chronic pain condition, microglia is activated. They change morphologically, increase in cell number, and alter the expression of neurotransmitter receptors, such as P2X4R, P2X7R and P2YR, in the spinal cord (Tsuda et al., 2005; Pocock and Kettenmann, 2007; Yang et al., 2015). Stimulation of microglial P2X4Rs evokes the synthesis and release of brain-derived neurotrophic factor (BDNF) (Ulmann et al., 2008; Trang et al., 2009). BDNF can enhance the excitability of the neurons in spinal dorsal horn through activating N-methyl-D-aspartic acid (NMDA) receptors. The mitogen-activated protein kinase (MAPK) is then activated and known to contribute to pain hypersensitivity and neuronal plasticity in pain.

Chronic pain poses a significant challenge in health care. For cancer-pain management, opioids are the cornerstone (Mercadante, 2010). For the treatment of chronic nonmalignant pain (CNMP), opioids are also widely used and incorporated into clinical pain management guidelines (Rosenblum et al., 2008). It is reported that μ opioid receptors (MOR) are primarily responsible for opioid analgesia and anti-nociceptive tolerance (Matthes et al., 1996). Meanwhile, MOR are the receptors that morphine binds to with the highest affinity (Pert et al., 1973). Morphine engagement of μ receptors in the central and peripheral nervous systems and gastrointestinal tract is accountable for its beneficial effects for pain relief as well as its deleterious effects of morphine tolerance.

**Figure 1:**
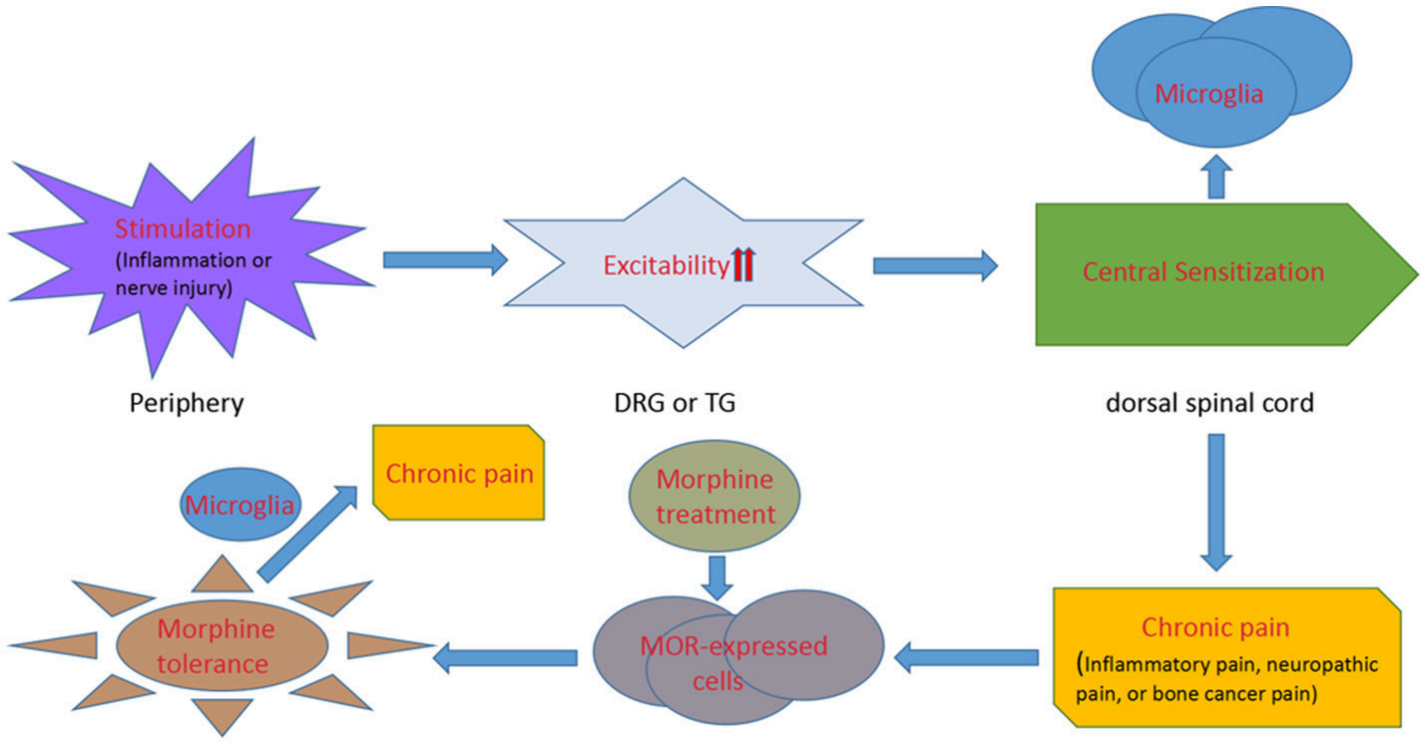
A diagrammatic presentation displaying the process from chronic pain to morphine tolerance. Inflammation or nerve injury stimulates the primary afferents of dorsal root ganglion or trigeminal ganglion neurons with an increasing excitability of the neurons. Then the electrical signals are transmitted to the spinal and medullary dorsal horns, where microglial cells are activated. The increasing expression of neurotransmitter receptors and pain pathways in microglia induces chronic pain. And, chronic morphine engagement of μ receptors in the central and peripheral nervous systems mediates morphine tolerance. Microglial cells are activated under the morphine tolerance circumstance, which finally exacerbates chronic pain.

In turn, morphine tolerance aggravates chronic pain. Opioid-induced hyperalgesia has been found in chronic pain patients (Mao et al., 1995; Ossipov et al., 2004). Moreover, chronic morphine exposure results in the activation of p38 MAPK (Cui et al., 2006) and a strong upregulation of the microglial markers CD11b and Iba1, as well as the ATP receptors P2X4 (Horvath et al., 2010) and P2X7 (Zhou et al., 2010) in spinal microglia, which intensifies the progress of pain. The relationship between chronic pain, microglia and morphine tolerance is illustrated in Figure 1.

## Regulatory mechanisms of MicroRNAs in chronic pain

Chronic pain could be divided into nociceptive pain (caused by inflamed or damaged tissue activating specialized pain sensors called nociceptors), and neuropathic pain (caused by damage to or malfunction of the nervous system) (Keay et al., 2000). In many types of cancers, the tumors have a strong predilection to metastasize to bones such as the vertebrae, ribs, hip, femur, and tibia (Coleman, 2006). Once cancer cells have metastasized to bone, they release algogenic substances that induce sensitization and activation of nerve fibers and cause nociceptive pain. Tumor growth in bone can also directly injury nerve fibers to generate a neuropathic pain (Mantyh, 2013).

### MicroRNAs and nociceptive pain

#### Intracellular miRNAs in nociceptive pain

miRNAs expressions in a pain model were first reported in 2007. The nociceptive pain was induced by injecting Complete Freund's Adjuvant (CFA) into the rat masseter muscle. Bai et al. showed the downregulation of miR-10a, miR-29a, miR-98, miR-99a, miR-124, miR-134, and miR-183 in TG, of which miR-124 was widely described as a key miRNA in pain-processing. The expression of miR-124 was decreased in the early state after the model was created, but was increased in the late state (Bai et al., 2007).

Kusuda et al. also used a CFA-induced inflammation model to investigate three preselected brain-specific miRNAs in mouse DRG, and spinal cord dorsal horn. RT-qPCR results showed markedly downregulation of miR-1, miR-16, and miR-206 expression. However, they were surprisingly upregulated in the spinal dorsal horn (Kusuda et al., 2011). Further research illustrated that miR-16 relieved CFA-induced chronic nociceptive pain of rats by targeting ras-related protein (RAB23) mRNA and inhibiting p38 MAPK activation (Chen et al., 2016). p38 MAPK expression and phosphorylation also inversely correlated to miR-125a-3p expression in orofacial nociceptive pain induced by CFA. When miR-125a-3p expression was down-regulated, the expression of p38 MAPK alpha was up-regulated in ipsilateral TGs at different time points after CFA injection. Furthermore, overexpression of miR-125a-3p significantly inhibited the p38 alpha mRNA level in ND8/34 cells. Taken together, miR-125a-3p is inversely correlated with orofacial nociceptive pain via modulating p38 MAPK (Dong et al., 2014).

The regulation mechanism of miRNAs in nociceptive pain and neuropathic pain can be similar or different. Study shows that miR-143 was downregulated in the DRG in animal model of peripheral inflammation (CFA injection) but not in a neuropathic pain model (transection of the sciatic nerve). It is suggested that miR-143 down-regulation in response to inflammatory cytokines could be responsible for the increase in versican V1/2 transcription and thereby contributed to the inflammation-dependent upregulation of versican in nociceptors (Tam Tam et al., 2011). Another miRNA, miR-134 has shown to be downregulated under both neuropathic conditions and nociceptive pains (Ni et al., 2013).

Studies show that miR-141-3p overexpression alleviated neuropathic pain development by targeting and inhibiting the high mobility group box1 (HMGB1) (Zhang et al., 2015) and it was also critical in CFA-induced chronic nociceptive pain both *in vitro* and *in vivo*. In the inflammation model, miR-141-3p expression was significantly upregulated with the increase in *HMGB1* gene expression. Meanwhile, thermal and mechanical pain thresholds were significantly decreased. While opposite effect was observed when administrating of miR-141-3p inhibitor (Shen et al., 2017).

miRNAs were shown to be dysregulated in nociceptive pain induced by other stimulation as well. In a spontaneous pain model (intraplantar of mice injected formalin), miR-124 expression was decreased in putative nociceptive spinal neurons and non-nociceptive DRG neurons (Kynast et al., 2013b). It is reported that MeCP2, a transcriptional regulator involved in nociceptive pain (Geranton et al., 2007, 2008) might be one of the target genes of miR-124 target gene. MeCP2 was upregulated following formalin or miR-124 inhibitor treatment, but downregulated following miR-124 mimic treatment. Intrathecal administration of a miR-124 mimic reduced the second phase of formalin-induced pain and chronic pain induced by carrageenan injection and peripheral nerve injury (Willemen et al., 2012). miR-155 and miR-233, centrally acting miRNA, were upregulated in the prefrontal cortices of rats upon carrageenan-facilitated facial inflammation (Poh et al., 2011). And in neonatal cystitis model (rats applied zymosan), miR-181a was shown to be involved in the modulation of spinal cord inhibition. These studies unmasked the existing new excitatory pathways to facilitate prolonged pain and hyperalgesia (Sengupta et al., 2013).

Epigenetic modification of miRNA promoter has shown to regulated miRNA expression in nociceptive pain. Ten-eleven translocation methylcytosine dioxygenase (TET) can catalyze DNA 5-hydroxylmethylcytosine (5hmC) in neurons of mammals. TET-mediated hydroxy methylation of miR-365-3p regulated nociceptive behavior. TET1 and TET3, which catalyzed 5hmC in the miR-365-3p promoter were significantly increased in the spinal cord of formalin-induced nociceptive pain mice. In addition, potassium channel, voltage-gated eag-related subfamily H member 2 (Kcnh2) as a target of miR-365-3p, played a critical role in nociceptive pain (Pan et al., 2016).

#### Circulatory miRNAs in nociceptive pain

Nociceptive pain could also be induced by miRNAs through unconventional mechanisms. let-7b was released from cultured DRG neurons to the serum followed by neuronal excitation with a variety of stimuli. Extracellular let-7b induces inward currents in rat DRG through coupling between toll-like receptor-7 and the nociceptive ion-channel transient receptor potential cation channel subfamily A. Intraplantar injection of let-7b elicited rapid spontaneous pain, while injection of its inhibitor reduced formalin induced spontaneous pain (Park et al., 2014). This report indicates that pain could be induced by circulatory miRNAs as well as intracellular miRNAs.

The functional targets of miRNAs were mRNAs translated to ionic channels (Kcnh2), transcriptional regulators (MeCP2), and signaling molecules (RAB23, TLR7, p38 MAPK). Both intracellular and circulatory miRNAs were involved in nociceptive pain and the circulatory miRNA let-7b contributed to nociceptive pain as well as intracellular miRNAs. The miRNAs reported to be involved in nociceptive pain in rodent models are summarized in Table 1.

**Table 1:** Characterized miRNAs in various conditions.

**miRNAs**	**Model**	**Region**	**Cell**	**Expression change**	**Up-regulation**	**Functional target**	**Therapeutic agent**	**References**
**INFLAMMATORY PAIN**								
miR-124	Rat CFA Mouse formalin Mouse carrageenan	TG DSC –	– Neuron –	Down (early) Up (late) Down N/D	N/D N/D Improved pain Improved pain	– – MeCP2 –	– – miR-124 mimic miR-124 mimic	Bai et al., 2007 Willemen et al., 2012
miR-1,-16,-206	Rat CFA	DRG dorsal spinal horn	Neuron Neuron	Down Up	N/D N/D	– –	– – –	Kusuda et al., 2011 Chen et al., 2016
miR-16	Rat CFA	DSC	Neuron	Down	Improved pain	RAB23	LV-miR-16	
miR-125a-3p	Rat CFA	TG	ND8/34 cell	Down	Improved pain	p38 MAPK	miR-125a-3p mimic	Dong et al., 2014
miR-143	Mouse CFA	DRG	Neuron	Down	N/D	versican V1/2	–	Tam Tam et al., 2011
miR-134	Rat CFA	DRG	Neuron	Down	N/D	μopioid receptor	–	Ni et al., 2013
miR-141-3p	Rat CFA	DSC	–	Up	Worsened pain	HMGB1	miR-141a-3p mimic	Shen et al., 2017
miR-155,-233	Mouse carrageenan	Prefrontal cortex	–	Up	N/D	N/D	–	Poh et al., 2011
miR-181a	Rat neonatal cystitis	DSC	N/D	Up	N/D	GABA_A_ receptor α1 subunit	–	Sengupta et al., 2013
miR-365-3p	Mouse Formalin	DSC	–	5hmC up	Worsened pain	Kcnh2	miR-365-3p AMO	Pan et al., 2016
let-7b	Rat Formalin	DRG	Neuron	–	Worsened pain	TLR7, cation channel subfamily A	Let-7b AMO	Park et al., 2014
**NEUROPATHIC PAIN**								
miR-1a-3p	Rat SNL Rat Axotomy Rat tibial SNI Rat sural SNI	DRG DSC DRG DSC DRG DRG	– – – – – –	Down Down UP Down Up Down	– – – – N/D N/D	– – – – – –	–	Kusuda et al., 2011 Norcini et al., 2014 Norcini et al., 2014
miR-21	Mouse SCI Mouse, rat axotomy Rat axotomy Rat CCI Nerve crush Rat SNL	Spinal cord DRG DRG DRG Sciatic nerve DRG	Astrocyte Neuron Neuron Neuron Axon Neuron	Up Up Up Up Up UP	N/D N/D N/D N/D N/D Worsened pain	– Sprouty2 – – – –	– – – – – miR-21 AMO	Strickland et al., 2011a Strickland et al., 2011b Yu et al., 2011 Sakai and Suzuki, 2013 Wu et al., 2011 Sakai and Suzuki, 2013
miR-7a	Rat SNL	DRG	Neuron	Down	Improved pain	Na _V_ β2	AAV-anti-miR-7a	Sakai et al., 2013
miR-96	Rat SNL Rat CCI	DRG DRG	Neuron Neuron	Down Down	N/D Improved pain	– Na _V_ 1.3	miR-96 mimic	Aldrich et al., 2009 Chen et al., 2014
miR-183	Rat SNL Rat CCI	DRG Spinal cord	– N/D	Down Down	Improved pain N/D	NaV1.3, BDNF mTOR/VEGF signaling pathway	AAV-miR-183	Lin et al., 2014 Xie et al., 2017
miR-30b	Rat SNL Rat SNL	DRG DRG	– neuron	Down Down	Improved pain Improved pain	Na_V_1.7 Na_V_1.3	miR-30b agomir miR-30b agomir	Shao et al., 2016 Su et al., 2017
miR-17-92	Rat SNL	DRG	Neuron	Up	Worsened pain	K_V_1.1, K_V_3.4, K_V_4.3 subunits	AAV-anti-miR-17-92	Sakai et al., 2017
miR-142-3p	Mouse SNL	DRG	–	Down	Improved pain	HMGB1	LV-miR-142-3p	Zhang et al., 2018
miR-19a	Rat CCI	–	–	Up	Worsened pain	SOCS1	–	Wang et al., 2015
miR-124	Rat SCI Mouse SCI Mouse intraplantar IL-1bin LysM-GRK2^+/−^	Spinal cord Spinal cord Spinal cord	– Neuron Microglia	Down Down Down	N/D N/D Improved pain	– – –	– – –	Nakanishi et al., 2010 Strickland et al., 2011a Willemen et al., 2012
miR-206	Rat CCI	DRG	–	Down	Improved pain	BDNF	miR-206 mimic	Sun et al., 2017
miR-195	Mouse SNL	DSC	Microglia	Up	Worsened pain	ATG14	–	Berliocchi et al., 2015
miR-128	Mouse SCI	Spinal cord	Microglia	Down	–	–	–	Yang et al., 2017
miR-218	Rat CCI	Spinal cord	Microglia	Up	Worsened pain	SOCS3	miR-218 AMO	Li and Zhao, 2016
miR-155	Rat CCI Rat CCI	Spinal cord Spinal cord	Microglia Microglia	Up Up	Worsened pain Worsened pain	SOCS1 SGK3	miR-155 AMO LV-anti-miR-155	Tan et al., 2015 Liu et al., 2015
miR-155	LPS	–	Microglia	UP	Worsened pain	RACK1	–	Yin et al., 2017
miR-200b/miR-429	Rat CCI	DSC	Microglia	Down	Improved pain	ZEB1	LV-miR-200b/miR-429	Yan et al., 2017
miR-146a	LPS	–	N/D	N/D	N/D	–	–	Yunta et al., 2012
miR-146a-5p	Mouse SNL	Spinal cord	Astrocyte	Down	Improved pain	TRAF6	miR-146a-5p mimic	Lu et al., 2015
miR-186-5p	Mouse SNL	Dorsal spinal horn	Neuron	Down	Improved pain	CXCL13	LV-miR-186-5p	Jiang et al., 2016
miR-103	Rat SNL	Spinal cord	Neuron	Down	Improved pain	Ca_V_1.2-α1, -α2δ1, β1 subunits	miR-103 mimic	Favereaux et al., 2011
miR-203	Rat Bilateral CCI	DSC	–	Down	N/D	Rap1a	–	Li et al., 2013
miR-23b	Mouse SCI	Spinal cord	GABAergic neuron	Down		NADPH oxidase 4	miR-23b mimic	Im et al., 2012
miR-500	Rat VRT	Spinal cord	GABAergic neuron	Up	Worsened pain	Gad1	miR-500 antagomir	Huang et al., 2016
**CANCER PAIN**								
miR-1a-3p	Mouse BCP	DRG	–	Up	Worsened pain	CLCN3	miR-1a-3p AMO	Bali et al., 2013
miR-34c-5p	Mouse BCP Mouse BCP	DRG DRG	– Neuron	Up Up	Worsened pain Worsened pain	– Ca_v_2.3	miR-34c-5p AMO	Bali et al., 2013 Gandla et al., 2017
miR-544-3p	Mouse BCP	DRG	–	Up	Unaffected	–	–	Bali et al., 2013
miR-483-3p	Mouse BCP	DRG	–	Down	Improved pain	–	miR-483-3p mimic	Bali et al., 2013
miR-370-3p	Mouse BCP	DRG	–	Down	N/D	–	–	Bali et al., 2013
miR-132	Mouse BCP	Spinal cord	–	Up	N/D	–	–	Hou et al., 2016
miR-124	Mouse BCP	Spinal cord	–	Down	Improved pain	Synaptopodin	miR-124 mimic	Elramah et al., 2017
**MORPHINE TOLERANCE**								
let-7a, c, g	Mouse opioid tolerance	Brain	–	Up	Worsened Opioid tolerance	μ opioid receptor	LNA-anti-let-7	He et al., 2010
miR-103,-107	Mouse opioid tolerance	Brain	Be(2)C cell	Up	–	–	–	Lu et al., 2014
miR-27a	Mouse opioid tolerance	Brain	Neuron	Down	N/D	Serpini1	–	Tapocik et al., 2016
miR-124	mouse chronic morphine treatment	Brain	Microglia, BMM	Up	–	p65, TRAF6	–	Qiu et al., 2015
miR-219	Mouse opioid tolerance Rat opioid tolerance	DRG spinal cord	– –	Down Down	Improved opioid tolerance Improved opioid tolerance	CaMKIIγ CaMKIIγ	miR-219 mimic LV-miR-219	Hu et al., 2016 Jian et al., 2017
miR-375	Mouse opioid tolerance	DRG	–	Down	Improved opioid tolerance	JAK2/STAT3 pathway	miR-375 agomir	Li et al., 2017
miR-365	Rat opioid tolerance	spinal cord	–	Down	Improved opioid tolerance	β-arrestin 2	LV-miR-365	Wang et al., 2016
miR-93	Mouse bone cancer	–	–	Up	Worsened opioid tolerance	Smad5	LV-anti-miR-93	Xiao et al., 2016
miR-338	Rat bone cancer	–	–	Down	Improved opioid tolerance	CXCR4	LV-miR-338	Mei et al., 2017

*N/D, not determined; CFA, complete Freund's adjuvant; LPS, lipopolysaccharide; BMM, marrow-derived macrophages; DRG, dorsal root ganglion; DSC, dorsal spinal cord; BCP, bone cancer pain; CCI, chronic constriction injury; SNL, partial sciatic nerve injury; SCI, spinal cord injury; SNI, spared nerve injury; VRT, ventral root transection; LV, lentivirus; HSV, herpes simplex virus; AAV, adeno-associated virus; LNA, locked nucleic acid; AMO, anti-miRNA oligonucleotides*.

### MicroRNAs and neuropathic pain

Lesions to the somatosensory nervous system frequently give rise to neuropathic pain, generally classified as either peripheral (originating in the peripheral nervous system) or central (originating in the brain or spinal cord) (Merskey and Bogduk, 1994; Torrance et al., 2006).

#### Dysregulation of miRNAs in peripheral neuropathic pain

DRG and TG neurons are the principal origins of neuropathic pain observed in nervous system injury, and their functional changes are involved in both the initiation and maintenance of chronic neuropathic pain (Devor, 2006). Upregulation and downregulation of miR-1a-3p is dependent on the types of peripheral nerve injury decided upregulation and downregulation of miR-1a-3p. miR-1a-3p expression was decreased in the DRG after partial sciatic nerve ligation, but increased after axotomy of the sciatic nerve, but in the dorsal spinal cord, it was decreased in both models (Kusuda et al., 2011). A study of 2014 showed tibial nerve injury upregulated its expression, while sural nerve injury downregulated miR-1a-3p expression in the DRG (Norcini et al., 2014). The report indicated that tibial nerve injury induced transient neuropathic pain, while sural nerve injury induced chronic neuropathic pain. The authors concluded that miR-1a-3p downregulation was involved in the development of chronic pain. On the other hand, Bastian et al. showed transfection of an miR-1a-3p mimic into cultured DRG neurons attenuated neurite outgrowth. This outcome indicates miR-1a-3p may be involved in recovery from nerve injury (Bastian et al., 2011).

miR-21 is one of the most well-characterized oncogenic miRNAs upregulated in almost all kinds of carcinoma cells (Selcuklu et al., 2009; Kumarswamy et al., 2011). It was upregulated in DRG neurons after various nerve injuries, including spinal cord injury (SCI) (Strickland et al., 2011a; Bhalala et al., 2013), axotomy (Strickland et al., 2011b; Yu et al., 2011), CCI (Sakai and Suzuki, 2013), nerve crush (Wu et al., 2011), and spinal nerve injury (SNI) (Sakai and Suzuki, 2013). Application of miR-21inhibitor suppressed the late phase of neuropathic pain (Sakai and Suzuki, 2013). Furthermore, miR-21 contributes to the functional recovery of DRG neurons from nerve injury (Strickland et al., 2011b; Zhou et al., 2015).

In DRG, miR-7a was closely related to the late phase of neuropathic pain sustainment through regulating neuronal excitability (Sakai et al., 2013). Small cell-sized neurons were considered to be nociceptive neurons in which miR-7a was drastically downregulated only in the late phase of neuropathic pain. In reverse, overexpression of miR-7a suppressed the maintenance of neuropathic pain. Furthermore, functional blockade of miR-7a caused neuropathic pain behaviors in normal rats. miR-7a targeted the β2 subunit of voltage-gated sodium channels to affected the cell surface expression of these channels, which decreased neuronal excitability (Isom et al., 1995; Lopez-Santiago et al., 2006). Voltage-gated sodium channels may be promising targets for neuropathic pain treatment (Devor, 2006). And upregulation of miR-7a may be an effective strategy for treating established neuropathic pain.

miR-182-96-183 cluster contains three adjacent miRNAs (miR-182, miR-96, and miR-183), which are consistently downregulated in pain conditions with distinct causes. The miR-182-96-183 cluster was downregulated in injured DRG and spinal cord neurons in neuropathic pain (Aldrich et al., 2009). In a chronic constriction injury (CCI) model, Chen et al. showed miR-96 was decreased within the ipsilateral DRG, but the Na_v_1.3 level was increased. Further examination suggested that miR-96 regulated neuropathic pain through inhibiting the expression of Na_v_1.3 in the DRG of CCI rats (Chen et al., 2014). As a member of the miR-182-96-183 cluster, miR-183 has also been illustrated to be critical in pain proceeding. Lin et al. showed that miR-183 was down-regulated in the DRG of partial sciatic nerve injury (SNL) rats. Meanwhile, overexpression of miR-183 significantly suppressed pain through targeting Na_V_1.3 and BDNF (Lin et al., 2014). Downregulating miR-183 expression promoted pain through activating the mTOR/VEGF signaling pathway in CCI rats (Xie et al., 2017). All the studies above indicate that miR-183 is a potential therapeutic target for neuropathic pain.

In addition to Na_V_ (Shao et al., 2016; Su et al., 2017), miRNAs modify voltage-gated potassium channels in neurons such as K_V_1.1, K_V_3.4 and K_V_4.3 in neuropathic pain. miR-17-92, a microRNA cluster with six distinct members (miR-17, miR-18a, miR-19a, miR-19b, miR-20a, and miR-92a) differentially targeting the 3′-UTR sequences of voltage-gated potassium channels and modulatory subunits, was presented to target potassium channels. Overexpression of miR-17-92 cluster contributed to pain through down-regulating potassium channels expression. Conversely, blockade of miR-17-92 alleviates mechanical allodynia in SNL rats (Sakai et al., 2017). miR-19a, a member of miR-17-92 cluster, was also reported to be upregulated in CCI though it targeted cytokine signaling 1 (SOCS1) (Wang et al., 2015). Thus, miR-17-92 shows great therapeutic potential as target in neuropathic pain treatment.

miRNAs also directly targeted proinflammatory cytokines mRNAs to modulate neuropathic pain behaviors. A significant reduction in miR-142-3p expression was observed in DRG of mice with SNL. Overexpression of miR-142-3p inhibited neuropathic pain and neuroinflammation through targeting high mobility group box 1 (HMGB1)(Zhang et al., 2018). This indicated that miR-142-3p may serve as a potential therapeutic target for neuropathic pain.

In peripheral nervous system, the dysregulation of miRNAs such as miR-1a-3p and miR-21 were dependent on the types of pain (SNL, SNI, axotomy, SCI, CCI or nerve crush). The included literatures also reported two microRNA clusters, miR-182-96-183 and miR-17-92, which mainly targets mRNAs translated to voltage-gated sodium and potassium channels. In general, miRNAs modulate pain-related behaviors by targeting the voltage-gated channels, the signaling pathway inhibitors and the proinflammatory cytokines.

#### Dysregulation of miRNAs in central neuropathic pain

##### MiRNAs in activated glial cells-induced neuropathic pain

Microglia modulated neurotransmission and neuroinflammation. And it has been increasingly recognized as major player in neuropathic pain (Ji et al., 2013). miR-124 was decreased in spinal neurons after spinal cord injury (Strickland et al., 2011a) and was shown to promote microglia quiescence (Ponomarev et al., 2011). Intraplantar IL-1β injection in spinal microglia of microglia/macrophage-specific GRK2 heterozygous (LysM-GRK2 437 +/) mice activated spinal microglia and developed prolonged inflammatory hyperalgesia concomitant with downregulation of miR-124 (Willemen et al., 2012). In addition, MeCP2, a transcriptional regulator involved in neuropathic pain, appears to be an important miR-124 target gene (Geranton et al., 2007, 2008). Intrathecal injection of an miR-124 mimic decreased expression of BDNF, a promoter of nociceptive transmission in the dorsal horn and a target gene of MeCP2, in intact mice.

Kusuda et al. (2011) showed miR-16 expression was increased in the DRG while it was decreased in the dorsal spinal cord after subcutaneous capsaicin injection. And miR-16 was also predicted to target BDNF. *BDNF* gene was also a direct target of miR-183(Lin et al., 2014) and miR-206(Sun et al., 2017), which were downregulated in neuropathic pain. And down-regulation of them was associated with an increasing in BDNF expression. miR-183 was also shown to be decreased in microglial cells and neurons in the spinal cord of CCI model (Xie et al., 2017). Thus, miRNAs were associated with BDNF signaling pathway in microglia.

Autophagy was found to be dysregulated in the spinal cord following peripheral nerve injury. Intrathecal injection of miR-195 inhibitor alleviated neuropathic pain and decreased proinflammatory cytokines (IL-1β and tumor necrosis factor-a) in primary spinal microglia. Interestingly, the analgesic effect could be impaired by the autophagy inhibitor, 3-methyladenine. Spinal nerve ligation decreased autophagic activity of primary spinal microglia, while miR-195 inhibitor increased autophagy (Berliocchi et al., 2015). As an important regulator of autophagy, ATG14 was identified to be a target for miR-195 (Obara and Ohsumi, 2011). Thus, miR-195 upregulation appears to contribute to neuropathic pain via inhibiting autophagy of microglia.

miR-128 was suggested to be downregulated in murine microglial BV2 cells (mouse microglia) in SCI model. Conversely, overexpression of miR-128 significantly promoted the conversion between M1 phenotype and M2 phenotype. Furthermore, miR-128 overexpression obviously decreased the concentration of TNF-α, IL-1β, and IL-6. Although the target of miR-128 and the exact mechanism was unknown, miR-128 overexpression significantly downregulated the expression levels of P38 and P-P38 (Yang et al., 2017).

Numerous signaling pathways are involved in microglia, and one of these is the Janus kinase (JAK)/signal transducer and activator of transcription 3 (STAT3) pathway, which was indirectly regulated by miR-218 in CCI rats. Overexpression of miR-218 down-regulated the expression of the suppressor of cytokine signaling 3 (SOCS3), leading to STAT3 activation. Moreover, downregulation of miR-218 suppressed microglia activation and STAT3 signaling activation in primary microglia. Thus, silencing of miR-218 may be a promising and novel treatment for neuropathic pain via inhibiting the activation of microglial cell STAT3 signaling and downstream proinflammatory genes of microglia (Li and Zhao, 2016).

miR-155 was significantly up-regulated in microglia both *in vivo* and *in vitro* (Yin et al., 2017) after neuropathic pain. Suppression of miRNA-155 attenuates neuropathic pain by regulating the expression of serum and glucocorticoid regulated protein kinase 3 (*SGK3*) (Liu et al., 2015) and SOCS1 (Tan et al., 2015) in CCI. Conversely, miR-200b and miR-429 were significantly decreased in isolated microglia and dorsal spinal cord. Zinc finger E box binding protein-1 (ZEB1), the target of miR-200b and miR-429, was greatly increased in CCI rats. Meanwhile, LV-miR-200b/miR-429 suppressed ZEB1 mRNA expression in rat microglial cells as well as neuropathic pain development (Yan et al., 2017).

As described above, in CCI mouse, the expression of miR-21 was increased in the spinal cord (Strickland et al., 2011a). Fluorescence illustrated that miR-21 was expressed in astrocytes. This implies that the activated astrocytes may contribute to neuropathic pain through miRNA.

Mónica et al. showed the dysregulation of miR-146a in the spinal cord after CCI and discussed the therapeutic potential of miR-146a in glia-mediated neuroinflammation (Yunta et al., 2012). As a result, a study published in 2015 illustrated that suppression of miR-146a-5p contributed to the maintenance of SNL-induced neuropathic pain through targeting TNF receptor associated factor-6 (TRAF6) which was expressed in spinal astrocytes. miR-146a-5p mimics attenuates neuropathic pain through inhibiting the activation of TRAF6/JNK pathway in the spinal astrocytes (Lu et al., 2015).

miR-186a-5p was shown to be downregulated in the dorsal spinal horn neurons after SNL. And C-X-C motif chemokine 13 (CXCL13), which is the direct target of miR-186a-5p drove spinal astrocyte activation and neuropathic pain via CXCR5. CXCR5 expression induced by SNL was required for the SNL-induced activation of spinal astrocytes and microglia. The study revealed a neuronal/astrocytic interaction in the spinal cord by which neuronally produced CXCL13 activates astrocytes via CXCR5 to facilitate neuropathic pain (Jiang et al., 2016).

##### MiRNAs in damaged nociceptive neurons-induced neuropathic pain

In spinal cord injury, some miRNAs were reported to be involved in key processes such as inflammation, cell death, regeneration, or gliosis (Nieto-Diaz et al., 2014). miR-124 play multiple roles in the spinal cord. Several studies showed that miR-124 was significantly decreased in putative nociceptive spinal neurons (Nakanishi et al., 2010; Strickland et al., 2011a). And intrathecal administration of miR-124 mimics reduced the second phase of formalin-induced pain (Kynast et al., 2013b).

The first well-characterized miRNA in neuropathic pain is miR-103(Favereaux et al., 2011). Its targets were three subunits (Ca _V_ 1.2-α1, -α2δ1, β1) (Fossat et al., 2010; Favereaux et al., 2011) of Ca_V_1.2L-type calcium channels, which underlie the long-term plastic changes in chronic neuropathic pain. miR-103 modulated their expressions to effect on calcium transients. In spinal dorsal horn neurons, miR-103 was downregulated in neuropathic pain. And intrathecal injection of miR-103 alleviated neuropathic pain through targeting the subunits of Ca_V_1.2.

In bilateral CCI model, miR-203 was downregulated in the dorsal spinal cord (Genda et al., 2013), but not in the DRG, anterior cingulate cortex or hippocampus (Li et al., 2013). Due to an undetermined target sequence, Rap1a was predicted to be a target of miR-203 *in vitro* (Li et al., 2015). This report showed that Rap1a expression was increased in the dorsal spinal cord, and former studies indicated that Rap1a which was a Ras family member involved in synaptic plasticity (Stornetta and Zhu, 2011) was shown to be increased in the spinal cord following nociceptive stimulation (Urayama et al., 1997). Therefore, miR-203 may be involved in neuropathic pain development.

miR-23b, was disclosed to play a crucial role in the amelioration of neuropathic pain in the injured spinal cord. The expression of miR-23b was decreased, while *NADPH oxidase 4 (NOX4)*, the target gene of miR-23b was up-regulated in neuropathic pain. miR23b infusion significantly improved paw withdrawal thresholds. Moreover, miR23b expression also alleviated neuropathic pain through protecting GABAergic neurons against ROS/p38/JNK-mediated apoptotic death (Im et al., 2012). Glutamate decarboxylase 67 (GAD67) modulates the function of GABAergic synapses in GABAergic neurons. Another miRNA, mir-500 was significantly increased and involved in the modulation of GAD67 expression via targeting the specific site of *Gad1* gene in neuropathic pain induced by ventral root transection (VRT). Moreover, using mir-500 antagomir rescued the GABAergic synapses and attenuated the sensitized pain behavior. So miR-500 may be a novel therapeutic option for neuropathic pain (Huang et al., 2016).

**Figure 2:**
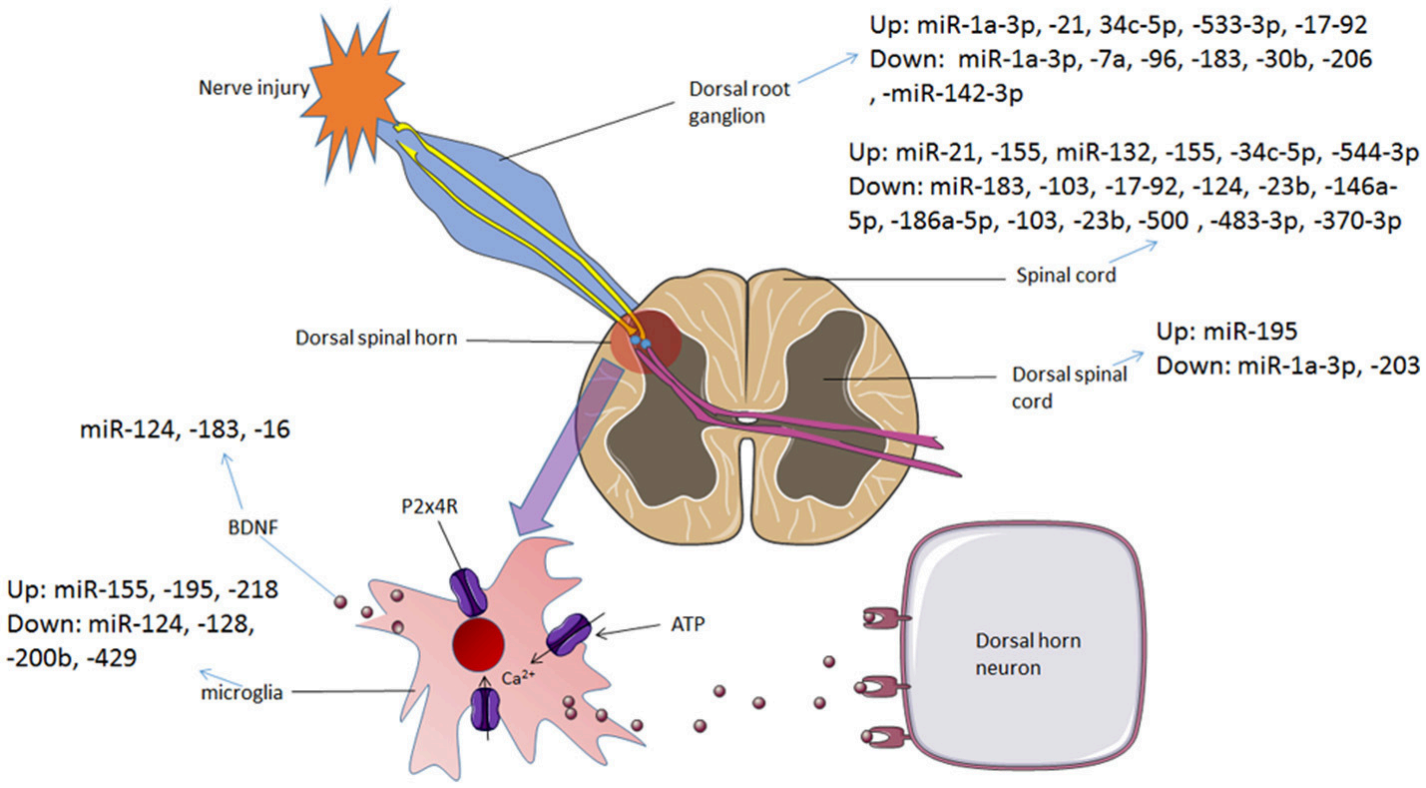
The dysregulation of miRNAs in different regions involved in neuropathic pain and bone cancer pain. In neuropathic pain and bone cancer pain, P2X4 receptors are activated and BDNF is released, which contributes to pain hypersensitivity. Various miRNAs are dysregulated during this process, from DRG to spinal cord and the microglial cells in the dorsal spinal horn.

Neuropathic pain in the central nerve system was mainly caused by activating glial cells and damage to the nociceptive spinal neurons in the spinal cord. The miRNAs promoted conversion between microglia phenotypes, modulated autophagy of microglia and altered the signaling pathways in the glial cells to effect on neuropathic pain. mRNAs of signaling molecules such as BDNF, SOCS3 and CXCL13 were direct targets of miRNAs both *in vivo* and *in vitro* glial cells. Voltage-gated calcium channels participate in neuropathic pain which was regulated by miRNAs. Interestingly, several miRNAs expressed in neurons improved synaptic plasticity which contributes to the amelioration of neuropathic pain. Table 1 presented the regulatory mechanisms of miRNAs in neuropathic pain reported in the studies. And the dysregulation of miRNAs in different regions involved in neuropathic pain is summarized in Figure 2.

### Bone cancer pain

The variety of miRNAs expressing in cancers have been intensively researched and investigated, while the miRNAs involved in cancer-related pain especially bone cancer pain is poorly understood. Bali et al. developed a bone cancer pain model followed by injecting tumor cells into the calcaneus bone, and showed that 57 miRNAs expressions were changed by more than 2.5-fold in the DRG, in which miR-1a-3p, miR-34c-5p and miR544-3p expression were increased after tumor cell implantation. Intrathecal administrating inhibitors of miR-1a-3p and miR-34c-5p suppressed the mechanical hypersensitivity, while miR544-3p inhibitors had no effect on bone cancer pain (Bali et al., 2013). Cav2.3, an antinociceptive Ca^2+^ channel in the peripheral sensory neurons, was a novel target for miR-34c-5p. Knocking down the expression of Cav2.3 specifically in DRG neurons led to hypersensitivity in mice (Gandla et al., 2017). Bali et al. also showed mice treated with a miR-370-3p-mimic developed significantly more tumor-induced mechanical hypersensitivity, and miR-483-3p-mimic inhibited the mechanical allodynia (Bali et al., 2013).

Chloride channel, voltage-sensitive 3 (CLCN3) which played roles in releasing synaptic vesicles in hippocampal glutamatergic neurons and regulating the amount of neurotransmitter, was expressed inpeptidergic and non-peptidergic putative nociceptive neurons of DRG (Guzman et al., 2014). The direct target of miR-1a-3p is CLCN3, and it was downregulated after tumor cell implantation. Knockdown of CLCN3 by siRNA injection enhanced cancer pain (Kusuda et al., 2011).

cAMP response element binding protein (CREB)-dependent gene expression plays an important role in central sensitization (Ma and Quirion, 2001). CREB-regulated transcription coactivator 1 (CRTC1) dramatically increases CREB-mediated transcriptional activity. miR-132 functions downstream from CREB/CRTC1 to mediate activity-dependent synaptic plasticity and in turn loops back to amplify CREB/CRTC1 signaling. p-CREB, CRTC1 and CREB-target genes (NR2B and miR-132) were up-regulated in a bone cancer pain model. And interrupting the positive feedback regulation between miR-132 and CREB/CRTC1can effectively relieve bone cancer pain (Hou et al., 2016). All above illustrates the positive feedback regulation between miR-132 and CREB/CRTC1 in the spinal cord contributes to the maintenance of bone cancer pain.

A recent study (Elramah et al., 2017) showed that miR-124 is down-regulated in the spinal cord in a mouse model of cancer pain. Synaptopodin (Synpo), a key protein for synaptic transmission, was a key component of the nociceptive pathways. miR-124 was an endogenous and specific inhibitor of Synpo, and intrathecal injections of miR-124 mimics normalized Synpo expression and completely alleviated cancer pain in the early phase of the cancer. The report suggests that miR-124 could be an efficient analgesic drug to treat cancer pain patients.

Few studies reported changes in miRNAs expressions of bone cancer pain due to the complicated regulatory mechanisms. Voltage-gated calcium and chloride channels as well as molecules of signaling pathways (Synpo) were targeted by miRNAs. And, it is important to emphasize that interruption to the positive feedback regulation between CREB/CRTC1 and miR-132 can effectively relieve bone cancer pain. The reported dysregulation of miRNAs involved in cancer pain is illustrated in Table 1 and Figure 2.

## Relevance of miRNA-based mechanisms in morphine tolerance

### Dysregulated miRNAs in brain

MOR agonists like morphine and fentanyl modulate miRNA expression (Wang et al., 1993; Wu et al., 2008; Zheng et al., 2010). Conversely, miRNAs are also capable of regulating MOR-1 expression (Wu et al., 2009, 2013). MOR 3′-UTR can be regulated by miR-23b in mouse. MOR-1 expression was markedly increased with deletion of miR-23(Zöllner et al., 2000). let-7 family also regulates the human MOR gene. In human neuroblastoma SH-SY5Y cells, let-7 inhibition induced a dose-dependent up-regulation of the endogenous MOR expression. While morphine increased let-7 expression *in vitro* and *in vivo* correlated with a downregulation of MOR. Injecting anti-let-7 into the brain attenuates opioid antinociceptive tolerance in mice (Atcheson and Lambert, 1994; He et al., 2010). Thus, let-7 is strongly associated with morphine tolerance. Other than let-7, miR-103 and miR-107 levels were also up-regulated with the increasing of MOR-1A protein in Be(2)C (human neuroblastoma cell) and striatum in morphine-tolerance mouse (Lu et al., 2014).

Serpin peptidase inhibitor clade I (Serpini1) mRNA which is a transcript known to be intricately involved in dendritic spine density regulation with chronic morphine's consequences is a target of miR27a. miR27a was found to positively regulate Serpini1 mRNA in multiple neuronal cell lines (N2A and N1E-115 neuroblastoma cells). And, Serpini1 knockout mice developed analgesic tolerance at a slower rate than wild-type mice (Tapocik et al., 2016). Thus, miR27a and Serpini1 played specific roles for morphine tolerance.

Long exposure of morphine maintained miR-124 expression in microglia, bone marrow-derived macrophages (BMM) and the mouse brain. miR-124 inhibited p65 and TRAF6-dependent TLR signaling, which causes immunosuppression of microglia. The study suggested that modulation of miRNAs is capable of preventing opioid tolerance-induced damage to microglia (Qiu et al., 2015).

### Dysregulated miRNAs in DRG and spinal cord

Calcium/calmodulin-dependent protein kinase II gamma (CaMKIIγ), has been identified to be a target of miR-219. In chronic morphine treatment mouse, miR-219 expression was downregulated with the increasing of CaMKIIγ and CaMKIIγ-dependent BDNF expression in DRG. In addition, morphine tolerance was markedly delayed by upregulating miR-219 expression or downregulating CaMKIIγ expression and was partially attenuated by BDNF via tyrosine receptor kinase B-Fc (Hu et al., 2016). Thus, miR-219 is associated with the development of morphine tolerance in mouse dorsal root. Another study demonstrated that miR-219-5p attenuated morphine tolerance by targeting CaMKIIγ in rats (Jian et al., 2017).

miR-365 was known to reverse morphine tolerance. Upregulation of miR-365 decreased the expression of β-arrestin 2 protein in the spinal cord, which alleviated the progressing of morphine tolerance. Thus, miR-365 upregulation provides a promising and novel approach for treatment of morphine tolerance (Wang et al., 2016).

Recently, a study demonstrated that miR-375 level was downregulated but the expression of Janus kinase 2 (JAK2) was upregulated in mouse DRG following chronic morphine treatment. And upregulation of miR-375 level could significantly hinder morphine tolerance by inhibiting the JAK2/STAT3 pathway (Li et al., 2017).

The expression of several miRNAs was dysregulated in bone cancer pain. miR-93 and its downstream target Smad5 were demonstrated to be correlated with morphine tolerance in bone cancer pain mouse model. miR-93 expression was increased and Smad5 mRNA expression was decreased after treating with morphine. Moreover, anti-miR-93 alleviated morphine tolerance. Overall, Up-regulation of miR-93 may contribute to the progression of morphine tolerance by targeting Smad5 in mouse bone cancer pain (Xiao et al., 2016). Morphine tolerance was remarkably suppressed in pLV-THM-miR-338 rats with lower expression of CXC chemokine receptor-4 (CXCR4). Although the specific mechanisms of miR-338 targeting of CXCR4 was unknown, miR-338 significantly influenced the inhibition of morphine tolerance (Mei et al., 2017).

Opioid antinociceptive tolerance contributes to chronic pain which implies alleviating morphine tolerance helps to reduce the maintenance of pain. Chronic morphine treatment induced miRNAs expressing more in the rodents' DRG, spinal cord and brain. In bone cancer pain, miR-93 and miR-338 were dysregulated by morphine tolerance. And the signaling pathways and signaling proteins such as JAK2/STAT3 pathway and BDNF which were targeted by miRNAs were also critical in pain conditions. The miRNAs both expressed in pain and morphine tolerance and targeted the same signaling molecules between chronic pain and morphine tolerance need to be taken seriously. Table 1 shows the regulatory mechanisms and therapeutic targets of miRNAs in morphine tolerance.

## Therapeutic potential of MicroRNAs in chronic pain and morphine tolerance

**Figure 3:**
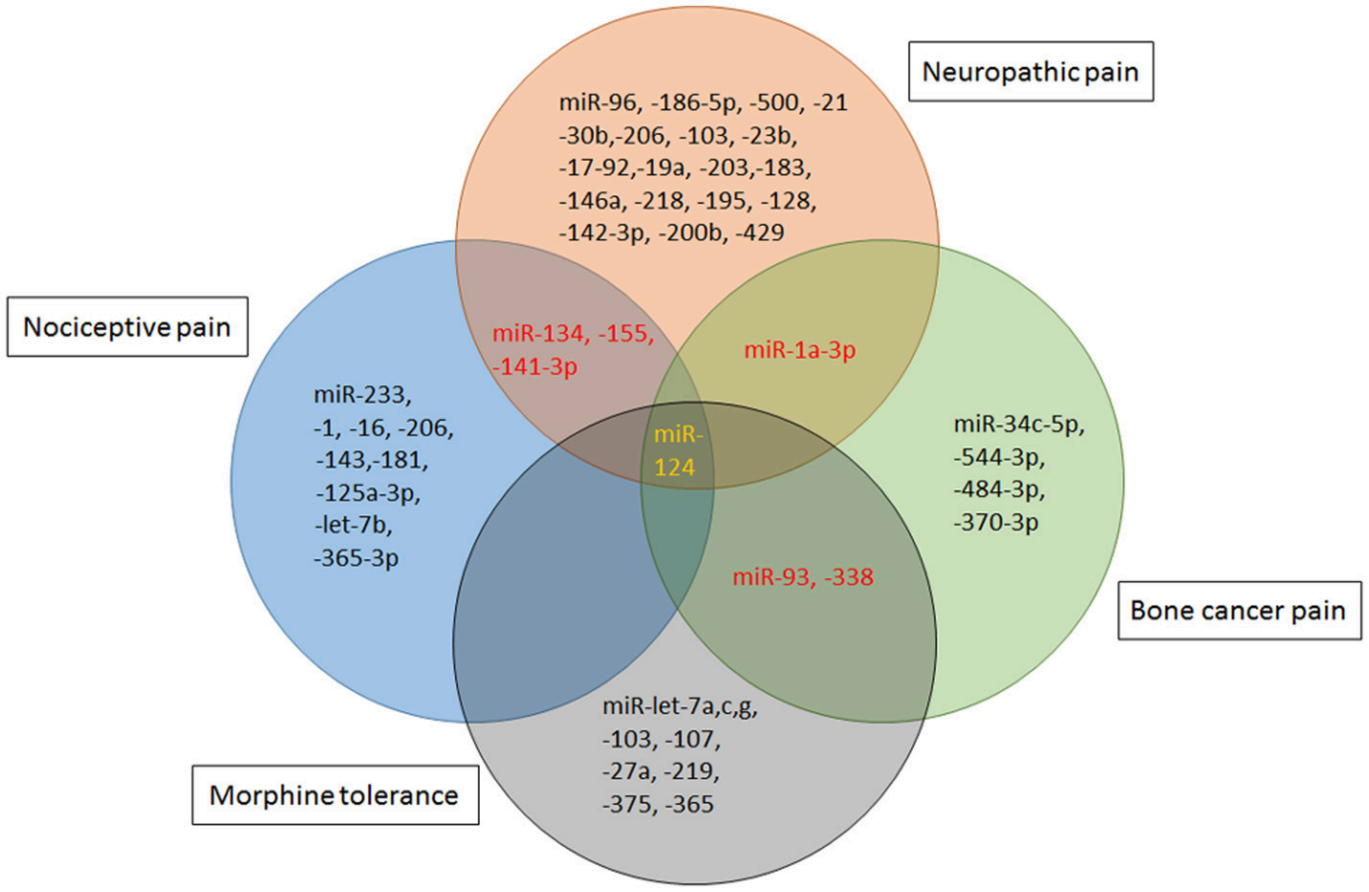
miRNA mimics and inhibitors administration in different rodent models of pain. Specific miRNAs that are altered in animal models of nociceptive pain [Formalin, complete Freund's adjuvant (CFA) or Carrageenan injection], neuropathic pain [chronic constriction injury (CCI), partial sciatic nerve injury (SNL), spinal cord injury (SCI), spared nerve injury (SNI) or ventral root transection(VRT)] and cancer-related pain [bone cancer pain (BCP)] have been identified as potential therapeutic targets. Schematic indicates that viral vectors [lentivirus (LV), herpes simplex virus (HSV), or adeno-associated virus (AAV)], stabilized locked nucleic acid (LNA) mimics, miRNA agomir or antagomir and anti-miRNA oligonucleotides (AMO) are therapeutic strategies that have been successfully reversed pain phenotypes.

Indeed, an exciting advance in pain biology has been more evident that miRNAs can modulate the expression of pain related mRNAs and proteins. Most miRNAs are nonoverlapping in different poor states, while modest overlap between altered miRNAs are reported in the literatures. miR-1a-3p and miR-132 were presented to be up-regulated in both neuropathic pain and bone cancer pain. While, miR-134,-155,-141-3p were dysregulated in nociceptive pain and neuropathic pain. And, dysregulation of miR-124 is manifested in all the above pain conditions as well as morphine tolerance (Figure 3). The high specificity between miRNA alterations contributes to the treatment of different types of pain and the side effects of pain therapy.

**Figure 4:**
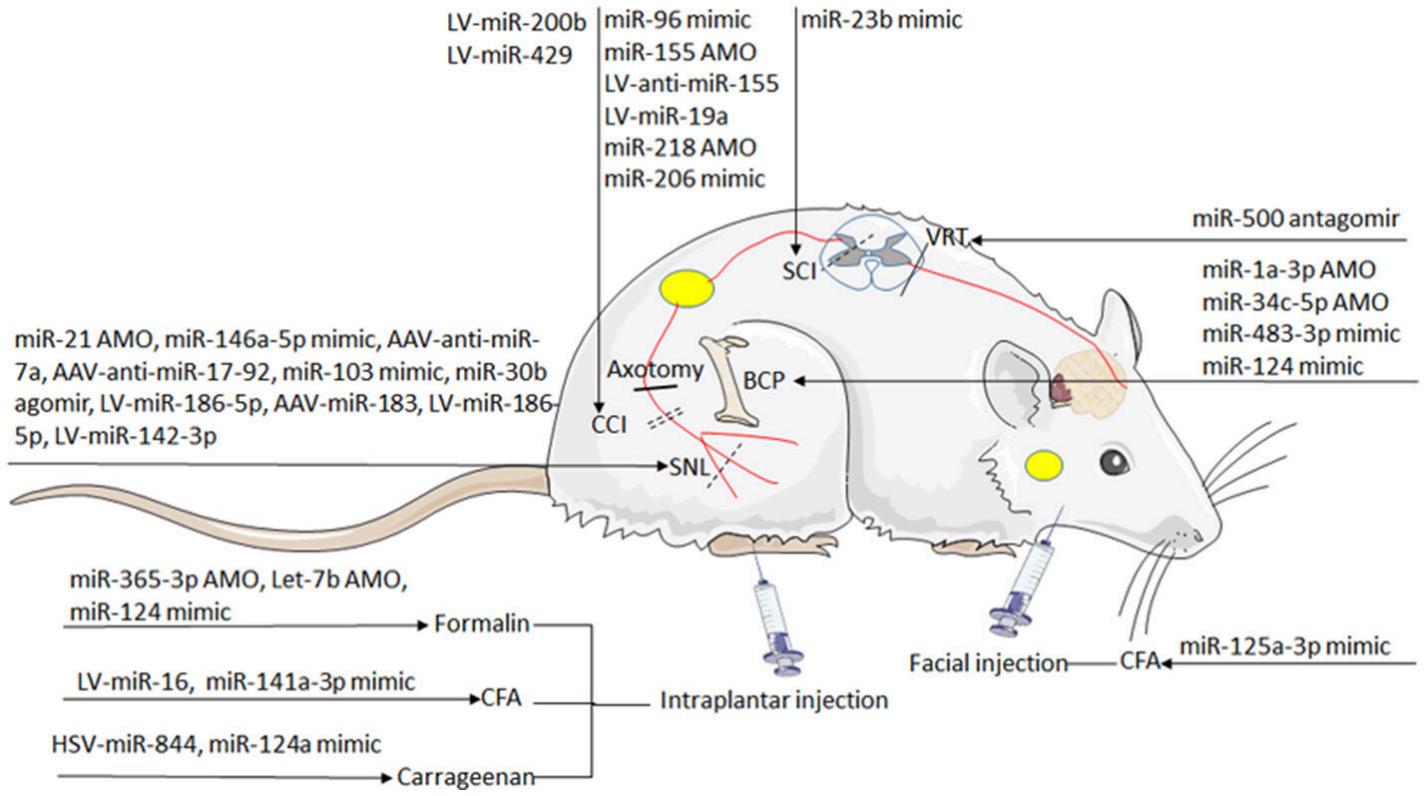
The overlaps between dysregulated miRNAs. The circles include miRNAs that have been mainly implicated in nociceptive pain, neuropathic pain, bone cancer pain, microglia and morphine tolerance. Venn diagram: overlapping regions indicate miRNA intersections (in common) with the reported abnormal states. miRNAs are indicated in black, red, and yellow if dysregulated in one, two, and four states, respectively.

Deciphering the impact of each miRNA on the post-transcriptional regulation is particularly critical. The direct targets of each miRNA under different conditions are presented in Table 1. Strategies based on miRNAs activity have been considered in cases of pathologic downregulation of miRNA expression. “therapeutic miRNA targeting” means “miRNA replacement therapy” as well as targeting and inactivating abnormally hyper-expressed miRNAs. Some studies successfully provided the proof-of concept for the role of specific miRNAs in influencing behavior of hyperalgesia and allodynia in rodent models. The effect of miRNA-induced attenuation of pain and morphine tolerance in rodent models has been demonstrated in Table 1 and Figure 4.

Using classical transfection methods such as lipofection or electroporation, miRNA mimics and inhibitors enter the cell. For the sake of increasing their half-life, some companies generated locked nucleic acids (LNA) to slightly modified the chemistry of the RNA oligonucleotides. In order to deliver miRNAs into the central nervous system, some studies use viral vectors such as adeno-associated virus (AAV), herpes simplex virus (HSV) and lentivirus (LV) to cross the blood brain barrier.

Although only a few miRNA therapeutic clinical trials is currently conducted (Bouchie, 2013; Janssen et al., 2013) and limited to Phase I studies for the treatment of cancer, the concept of “therapeutic miRNA targeting” has attracted great interest and multiple future therapeutics are under developments. For example, miR-16 mimics (TargomiRs) has been used in patients failing to respond to standard therapy (Van et al., 2015). Because miR-16 is also involved in controlling RAB23 expression in CFA induced nociceptive pain (Chen et al., 2016), future applications of miRNA technology might be used to decrease RAB23 expression and ameliorate patient discomfort of pain conditions.

## Directions of future research on miRNAs

### In cancer-related pain

Causes of cancer pain are multifactorial and complex and are likely to vary with tumor-related and host-related factors and processes, and the pathophysiology is poorly understood (Chwistek, 2017). Several literatures provide data on miRNAs in bone cancer pain animal models. In addition, the mechanism of miRNAs during progress of cancer pain is not explicit. Further research is needed to elaborate the regulatory mechanisms of miRNAs in bone cancer pain, and miRNAs would be prognostic and diagnostic biomarkers and potential new drug targets for chronic pain including cancer pain (Kress et al., 2013; Kynast et al., 2013a).

### In purinergic signaling network

The involvement of P2 receptors (P2X3R, P2X4R, and P2X7R) in mediating and maintaining nociceptive sensitivity, as well as neuropathic and nociceptive pain, has been ascertained in different studies (Kobayashi et al., 2011; Bele and Fabbretti, 2015; Burnstock, 2016). And miRNAs have been shown to be capable of modulating the purinergic signaling network. In a rat model of streptozotocin-induced diabetes, miR-9 modulated P2X7 receptor-mediated signaling to pain sensation. miR-9 and calcium homeostasis modulator 1 (CALHM1) were upregulated in spinal dorsal horn neurons. Nevertheless, none literature publishes data of miRNA in relation to P2X4R. Moreover, microglial P2X4R are central players in the pathogenesis of tactile allodynia in neuropathic pain (Inoue, 2017). Owing to this, future research is necessary to demonstrate the relation between miRNA and P2X4R in chronic pain.

### In microglia related to morphine tolerance

Despite numerous miRNAs are demonstrated to be aberrantly expressed in chronic pain and morphine tolerance, few miRNAs were overlapped. In this article, only one miRNA (miR-124) was dysregulated in microglia both in chronic pain and morphine tolerance. Since microglia is critical in aggravating chronic pain when morphine tolerance develops, miR-124 might be an interesting target for future studies. Although miR-93 and miR-338 were both dysregulated in chronic morphine-treatment bone cancer pain, but whether they were expressed in microglia is unknown. No doubt future studies are likely to provide better insights into miRNAs in microglia of morphine tolerance to reveal the function of miRNAs in the aggravation of chronic pain caused by morphine tolerance.

### miRNA replacement therapy

Anti-miRNAs and miRNA-mimics has been frequently applied in animal models of chronic pain to accomplish diagnostic or therapeutic utilization. While the miRNA-therapy in human is warranted to be adequately studied. As described above, miR-16 mimics has been performed in patients failing to respond to standard therapy. As a result, the highly specific miRNAs altered in different poor conditions with the same mechanisms are willing to be especially studied.

## Conclusions

In animal pain models such as rodent spinal nerve ligation, CFA-induced inflammation and bone cancer pain, wide-spread miRNA modulation is observed. This review summarized the mechanisms of miRNAs regulation in different pain conditions and morphine tolerance. The dysregulation of miRNAs in glial cells was highlighted due to the critical role of these cells in chronic pain. In addition, the use of anti-miRNAs and miRNA mimics as potential therapeutic agents were shown to be effective in reversing pain and morphine tolerance.

The roles of miRNAs play in chronic pain are likely to vary depending on the exact causes of chronic pain. Further researches focusing on the understanding of key miRNAs in regulating chronic pain are needed to validate these miRNAs as potential therapeutic target. For examples, understanding the roles of miRNAs play in purinergic signaling network and the regulation of P2 receptors is of great importance. Since microglia is critical in aggravating chronic pain when morphine tolerance develops, miR-124 might be an interesting target for future studies as it was dysregulated in microglia both in chronic pain and morphine tolerance. For future chronic pain and morphine tolerance treatment, anti-miRNAs and miRNA-mimics may prove to be a valuable option once the link between specific miRNAs and chronic pain or morphine tolerance is well established.

## Author contributions

All authors listed, have made substantial, direct and intellectual contribution to the work, and approved it for publication.

### Conflict of interest statement

The authors declare that the research was conducted in the absence of any commercial or financial relationships that could be construed as a potential conflict of interest.
